# Self-Transcendence as a Buffer Against COVID-19 Suffering: The Development and Validation of the Self-Transcendence Measure-B

**DOI:** 10.3389/fpsyg.2021.648549

**Published:** 2021-10-06

**Authors:** Paul T. P. Wong, Gökmen Arslan, Victoria L. Bowers, Edward J. Peacock, Oscar Nils Erik Kjell, Itai Ivtzan, Tim Lomas

**Affiliations:** ^1^Department of Psychology, Trent University, Peterborough, ON, Canada; ^2^Department of Psychological Counseling and Guidance, Mehmet Akif Ersoy University, Burdur, Turkey; ^3^Centre for Wellbeing Science, Melbourne Graduate School of Education, University of Melbourne, Melbourne, VIC, Australia; ^4^Graduate College, Saybrook University, San Francisco, CA, United States; ^5^Independent Researcher, Peterborough, ON, Canada; ^6^Department of Psychology, Lund University, Lund, Sweden; ^7^Department of Psychology, Naropa University, Boulder, CO, United States; ^8^Department of Psychology, University of East London, London, United Kingdom

**Keywords:** self-transcendence, meaning, COVID-19, existential positive psychology (PP2.0), existential psychology, positive psychology, mature happiness, wellbeing

## Abstract

The age of COVID-19 calls for a different approach toward global well-being and flourishing through the transcendence suffering as advocated by existential positive psychology. In the present study, we primarily explained what self-transcendence is and why it represents the most promising path for human beings to flourish through the transformation of suffering in a difficult and uncertain world. After reviewing the literature on self-transcendence experiences, we concluded that the model of self-transcendence presented by Frankl is able to integrate both of the characteristics associated with self-transcendence. Afterward, we discussed how the self-transcendence paradigm proposed by Wong, an extension of the model by Frankl, may help awaken our innate capacity for connections with the true self, with others, and with God or something larger than oneself. We presented self-transcendence as a less-traveled but more promising route to achieve personal growth and mental health in troubled times. Finally, we presented the history of the development and psychometrics of the Self-Transcendence Measure-Brief (STM-B) and reported the empirical evidence that self-transcendence served as a buffer against COVID-19 suffering. The presented data in the current study suggested that the best way to overcome pandemic suffering and mental health crises is to cultivate self-transcendence.

**Self-Transcendence as a Buffer Against COVID-19 Suffering: The Development and Validation of the Self-Transcendence Measure-B**

“The essence of life is to serve others and do good.”- Aristotle

## Introduction

The human costs of the pandemic in terms of mortality, morbidity, mental health conditions, and direct economic losses are staggering (Cutler and Summers, 2020; Graham, 2020). Even with three vaccines, the Centers for Disease Control and Prevention (CDC) still warns of a 4th wave of COVID-19 (Soucheray, 2021). The pandemic may persist for years, according to the Chief Public Health Officer of Canada (Aiello, 2020). The pandemic has destroyed countless businesses and careers. It becomes more challenging to rebuild an own career or start a new one. Therefore, an individual needs to be equipped with the mental toughness to excel in whatever one pursues. The positive illusion of focusing only on the positive side of life will not carry one very far in a world full of cut-throat competition and the human evils of deception and discrimination.

For people who have lost everything, the struggle may seem like a futile attempt to endlessly push a rock uphill (Camus, 1942/1955). The pandemic fatigue may turn into pandemic burnout (Millard, 2021). The universality of suffering is a fact that we can no longer deny or avoid in the age of COVID-19. The natural tendency to avoid or escape from the pain of life may create more problems for us. Paradoxically, the greatest discovery of dialectical psychology is radical acceptance, the idea that to embrace and make the best use of all of one's pain for a better life is a better alternative to finding healing and flourishing.

However, our optimistic view (Wong, 2020a, 2021a,e) is based on empirical evidence (Bethelmy and Corraliza, 2019; Lin et al., 2021; Worth and Smith, 2021; Yaden and Newberg) regarding the power of self-transcendence, which comes from altruism (Feigin et al., 2014), religious faith (Koenig, 2009), meaning (Wong, 1998), and the feeling of awe (Yaden et al., 2017). According to Wong (2021b), the worst of times is also the best of times, but we cannot celebrate the best without overcoming and transcending the worst. This sums up the power of self-transcendence, which achieves the dual purpose of transforming suffering into resilience and motivating us to pursue the highest ideals. This vertical dimension of self-transcendence is often neglected because current psychology research tends to focus on the horizontal dimension of self-actualization, pro-social behaviors, and the emotion of awe.

In sum, when probably understood, self-transcendence represents what is good and noble about humanity. It is like a symphony celebrating the heroic efforts of those who sacrifice themselves in order to lift others above their suffering to a higher ground of faith, hope, and love. More importantly, it has the power of inspiring countless others to transcend their petty, egotistic concerns and serve something greater than themselves. However, this mega shift demands more than individual endeavors. It also requires a collective effort to co-create a more just and compassionate society.

At this crucial juncture of history, it is advisable to honestly and deeply examine our cultural beliefs and our own existential crises. In this article, we intended to explain and demonstrate empirically why self-transcendence can not only buffer us against COVID-19, but also transform us from a self-centered and self-absorbed existence to a self-transcendent and fulfilling life.

Cushman's (1990) social-historical analysis on “the empty self-phenomenon” is still relevant to the current meaning crisis. He pointed out that broad historical forces, such as industrialization, urbanization, and secularism, have shaped Western culture and influenced the predominant psychological view of human beings as bounded, autonomous, masterful, but with empty selves. America is one of the richest countries, but it is also one of the emptiest ones because Americans try to fill their inner emptiness with the consumption of products, drugs, psychotherapy, or happiness coaches. Such efforts are self-defeating because our spiritual yearning for meaning can only be filled by constitutive goods, which are intrinsic to human existence, such as friendship, justice, and prosocial behavior.

The main argument in Cushman's study is that “in a world sorely lacking in community and tradition, the most effective healing response would be to address those absences through structural societal change by reshaping political relationships and cultural forms and re-establishing the importance of their transmission” (p. 607). He raised the important question, “Could psychology now become a helpful force, assisting in the development of a perspective on the masterful, bounded self in opposition to the current system? Given the history of the Western self and the role of psychology within that history, it is doubtful” (p. 609).

In view of Cushman's analysis, our current mental health crisis is partly one of our own making, with the misguided Western conception of human beings as self-contained masterful individuals who are entitled to feel happy every day by consuming instrumental rather than constitutive goods. Constitutive goods are natural and intrinsic goods, such as connections with the self, with others, and with God because of our human nature, according to the self-transcendence paradigm of Wong (2020a, 2021a). More importantly, we hope that this push for a change of direction in psychology will also drive the public dialogue toward how to restore civil virtues and spiritual values in our culture, such as responsibility, cooperation, altruism, reverence for life, and benevolence.

Self-transcendence is important for psychology and society because it is a promising way to balance self-interest with social interest. Consistent with Cushman's analysis, mainstream psychology still focuses on the scientific study of different aspects of the self as a masterful individual, such as the internal locus of control, self-efficacy, self-control, self-esteem, signature character strengths, and achievement. However, during difficult times, such as this pandemic, survival and flourishing depend on our ability to transcend adversity and selfishness by making the necessary sacrifices for the common good. There is an urgent need for public awareness of the importance of “we” over “me” in times of national crises.

Paradoxically, the power of self-transcendence in contributing to well-being, resilience, and the richness of our lives seems to come from embracing and transcending suffering, vis-à-vis from losing ourselves in the beauty and goodness around us to sacrificing immediate gratification in pursuit of the long-term worthy life goal to serve the common good. In the final analysis, it appears that developing the capacity to endure and transcend our misfortunes, trauma, sufferings, and fears over and over again in order to serve something or someone is more important for surviving and thriving, especially in the era of COVID-19. The lifelong research of the first author and clinical practice support this proposition. This is the core message of this study.

### What Is Self-Transcendence

According to the American Psychological Association (APA), self-transcendence is “the state in which an individual is able to look beyond himself or herself and adopt a larger perspective that includes concern for others. Some psychologists maintain that self-transcendence is a central feature of the healthy individual, promoting personal growth and development (first described by Viktor E. Frankl) (American Psychological Association, 2020a).” Literally, self-transcendence refers to that which goes beyond our own limitations and difficulties in life experience in order to serve or become connected with something greater. According to Ackerman (2021), self-transcendence is experienced when there is a “realization that you are one small part of a greater whole, and act accordingly.” This humanness of being bound by all kinds of constraints can only be unbound through the transcendental realm (Elmer et al., 2003).

The transcendence principle encompasses nature, science, religion, politics, culture, or anything that is greater or beyond human experiences (van Deurzen, 2014). Transcendence also generally refers to the complex emotions that arise from a sense of unity with other people, nature, and God, such as awe and selflessness (Keltner and Haidt, 2003; Mikulak, 2015; Yaden et al., 2017).

### Self-Transcendence as a Religious or Spiritual Experience

Self-transcendence involves the motivation to experience something sacred and beyond the daily mundane experience. Human existence is ordinary and material until we can perceive and take hold of the part of us that seeks out the realm of sacred experience larger than the self (Mayseless and Russo-Netzer, 2017). These self-transcendent sacred moments add some deeper spiritual meaning to our lives.

Self-transcendence is generally related to religious experiences. For example, William James (1902/2004) wrote: “The only thing that religious experience, as we have studied it, unequivocally testifies to is that we can experience union with something larger than ourselves and in that union find our greatest peace.” van Deurzen (2014) pointed out that transcendence can also be related to polytheism:

“Transcendence is experienced as related to the many different gods and divine representations that need to be appeased. Many clients hold such hidden views. They say prayers to the various deities, icons, or saints they believe in and feel they will only be able to surpass their problems if the gods are favorable to them. Polytheistic beliefs often go with a strong sense of community and community support is crucial for transcendence to become possible.”

Similarly, Frey and Vogler (2019) concluded that, according to research in the humanities and social sciences, individuals who can locate themselves in a larger or broader perspective, whether it is within the family, community, or religious or spiritual groups, often experience greater happiness, meaning, and virtue. More importantly, this finding is relevant to all major religions, both Eastern and Western.

### Self-Transcendence as an Emotional Response of Awe

The self-transcendent emotion refers to a category of emotions such as awe, love, elevation, and appreciation, among others, that connect people in social relationships (Stellar et al., 2017).

Awe is a natural emotional response to something spectacular or sacred, such as a glorious sunset or an ancient temple, which may be considered as a deep existential-spiritual response (Schneider, 2009, 2011). According to a study by Keltner and Haidt (2003) on cognitive interpretation, awe tends to arise from a perception of vastness and a cognitive need to accommodate our own perception into existing mental schemas.

The importance of this transcendental emotion, specifically for well-being, has received increasing empirical attention (Yaden et al., 2017; Allen, 2018). For example, Bethelmy and Corraliza (2019) reported that various recent studies have demonstrated the power of nature to induce the transcendental emotion of awe. They developed an instrument to measure sublime emotion toward nature, which includes the feeling of awe and inspiring energy. More recently, Clewis et al. (2021) discovered that there is much overlap between awe and sublime feelings, suggesting that these two pieces of literature could inform one another. In terms of application, Passmore and Holder (2016) reported that a 2-week nature-based well-being intervention increased transcendental benefits such as positive affect, elevating experiences, a general sense of connectedness (to other people, to nature, and to life as a whole), and prosocial orientation as compared with the human-built and control groups.

According to the self-transcendence model described later, there are at least seven ways to boost the emotions of awe. We can stand in awe of the following phenomena:

1. The power of life forcing us to grow and bear fruits against all odds.
2. The wisdom of human mind to probe into the mystery of life.
3. The virtue of love in sacrificing the self for others.
4. The magic of gratitude to fill our hearts with happiness.
5. The invincible courage to stand up against evil.
6. The unwavering faith in creating a better future.
7. The unspeakable joy of losing oneself to become a part of something greater, e.g., nature or the Creator.

### Self-Transcendence as Altruistic and Prosocial Behavior

Self-transcendence has both a vertical and horizontal dimension. We can transcend upward toward God, nature, or an invisible spiritual realm, and we can also transcend toward others by serving and connecting with them. We proposed that our innate self-transcendence tendency can account for altruistic, helping, and prosocial behaviors. This tendency can be reinforced by the intrinsic feeling of good from doing good (Mruk, 2018) and the positive effect of connecting with others, thus meeting our need for companionship and social and emotional support (Meek, 2012). Recently, some researchers found that the natural transcendental emotional response to some powerful stimuli is sufficient to make one forget our own “small self” and pay attention to others or engage in prosocial behavior (Piff et al., 2015; Guan et al., 2019; Li et al., 2019).

In contrast to egotism, altruism is motivated by our desire to increase the welfare of another person, even when it may cost something to ourselves (Bartlett and DeSteno, 2006). It is the kind of prosocial behavior that benefits others without any expectation of return (Eisenberg and Miller, 1987; Feigin et al., 2014). There is increasing research evidence that the natural emotional response of awe is sufficient to make one forget their own small self and self-interest and, instead, pay attention to others or engage in prosocial behavior (Keltner and Haidt, 2003; Piff et al., 2015; Li et al., 2019).

Volunteering is another form of prosocial behavior, which is very relevant to retirees today because it enables them to stay connected and make some contribution to society and, in return, contributes to their health and meaning in life (Wilson, 2000; Midlarsky and Kahana, 2007). Recent research has confirmed that such acts of kindness contribute to own well-being (Curry et al., 2018; Hui et al., 2020).

### Self-Transcendence as Virtue and Value

According to the study of Peterson and Seligman (2004), transcendence encompasses several character strengths such as an appreciation for beauty gratitude, hope, humor, and religiousness (having a solid belief about a higher purpose and meaning of life); it represents one of the six virtues. Although they do not make explicit references to God or religion, Peterson and Seligman (2004) recognized the universality of religion and spirituality: “Although the specific content of spiritual beliefs varies, all cultures have a concept of an ultimate, transcendent, sacred, and divine force” (p. 601).

The large-scale studies by Sortheix and Schwartz (2017) among European countries found that self-transcendence was positively related to subjective well-being. More importantly, they did not find any support for the expectation that self-transcendence might also be detrimental to subjective well-being because self-transcendence often entails investing more in the well-being of others than in their own.

### Self-Transcendence as Personal Growth

Humanism is also based on the principle of self-transcendence (van Deurzen, 2014). According to humanistic psychology, self-transcendence is a growth motivation to connect with others. According to DeRobertis and Brand (2019):

“The purpose of the self-transcending motivational tendency is to relate to things and others in the most meaningful and profound manner, overriding (without necessarily eliminating) concern for own enjoyment or self-interest.”

Wong (2016a) described self-transcendence as a paradoxical way toward personal growth. In other words, the best path toward self-actualization and personal growth is through transcending our limitations toward the greater good. His approach is based on meaning-seeking model proposed by Wong (2016b), which will be explained in detail later.

Maslow (1971) comes to a similar conclusion. To him, transcendence represents the highest level of human development and the higher consciousness of being connected. Thus, those who achieve self-transcendence have experienced peak experiences and satisfied their being-needs such as wholeness (unity), perfection (balance and harmony), justice (fairness), autonomy (self-sufficiency), and meaningfulness (values):

“Transcendence refers to the very highest and most inclusive or holistic levels of human consciousness, behaving and relating, as ends rather than means, to oneself, to significant others, to human beings in general, to other species, to nature, and the cosmos” (Maslow, 1971, p. 269).

On the other hand, Frankl argued that self-transcendence was merely a by-product of pursuing self-transcendence. Wong (2005) provided a similar but more detailed critique of Maslow's hierarchical model. However, Kaufman (2020) is correct in arguing that there is no real conflict between self-actualization and self-transcendence because one moves freely from one state to another in a fluid manner. Actually, everyone begins with self-actualization, as we desire to develop our potentials and become our best selves; however, this can only happen when we are awakened to our need for b-values of being-values, such as goodness, rightness, justice, and benevolence, which are the kind of values that characterize self-transcendence. No purposes are equal. If the life goal of an individual is egotistic and worldly, success may result in them ruining their own lives (Holiday, 2016).

The study by Rogers (1961) on organismic valuing theory is also moving toward the direction of self-transcendence as evidenced in the study by Kaufman (2020) on the new science of self-actualization. Likewise, Maurer and Daukantaite (2020) reviewed a great deal of empirical evidence supporting the connection between the organismic valuing theory of personal growth and self-transcendence. In a qualitative study of highly self-transcendent individuals, Reischer et al. (2021) confirmed that such individuals tended to narrate their lived experiences as spiritual journeys of humanistic growth toward self-transcendence during the later middle age.

From the psychodynamic perspective, Dobson (2015) reported that self-transcendence represents personal growth toward spiritual and moral maturity:

“For Jung as well as for Kohut, one's maturation into a whole self, one's hoped-for completion of the task of individuation is a transcendent, transpersonal act of great moral responsibility” (p. 9).

### Self-Transcendence Contributes to Mature Happiness

Life is full of dilemmas, paradoxes, contradictions, and suffering. We need to face life in its totality with the existential courage to face suffering and death (Van Tongeren and Van Tongeren, 2020): “Courage is the universal self-affirmation of one's Being in the presence of the threat of non-Being” (Tillich, 1952, p. 163). The only way we can find ways to transcend adversity is to learn how to muster enough courage to face the stress and negative emotions of each day.

van Deurzen (2014) provided a very useful survey of major existential philosophers to make the point that the single-minded pursuit of happiness as the end goal of life is doomed to fail. She began by pointing out that it is not possible to think of the positive without thinking of the opposite side: “Happiness and unhappiness are twins that grow up together”. Therefore, there is a need to transcend and integrate all the contractions and oppositions in life, according to the dialectics of Hegel on synthesis from the thesis and antithesis. Greater truths and integrations in life and in research, can only come from such a synthesis. She also cited the notion of Sartre on re-inventing oneself or re-defining a situation so as not to get stuck: “Man is characterized above all by his going beyond a situation and by what he succeeds in the making of what he has been made”. Therefore, she eloquently pointed out the impossibility of personal growth and mature happiness without transcending the unavoidable and inescapable paradoxes and sufferings in human existence:

“The paradox of life is that only if we accept both aspects of these oppositions and contradictions that we can transcend our difficulties and find new and more creative ways to encompass the whole span of human ability and challenge.

It is only when we are willing to face death and pain that we can live life to the fullest, instead of worrying ourselves sick and trying to be healthy and wealthy and comfortable all the time, which leads to a life lived in fear.

It is only when we allow ourselves to notice our weakness and vulnerability that real strength is found instead of us covering up our doubts by narcissistic pretense or giving in to our fragility by self-destructive denial of ourselves.”

**Figure 1:**
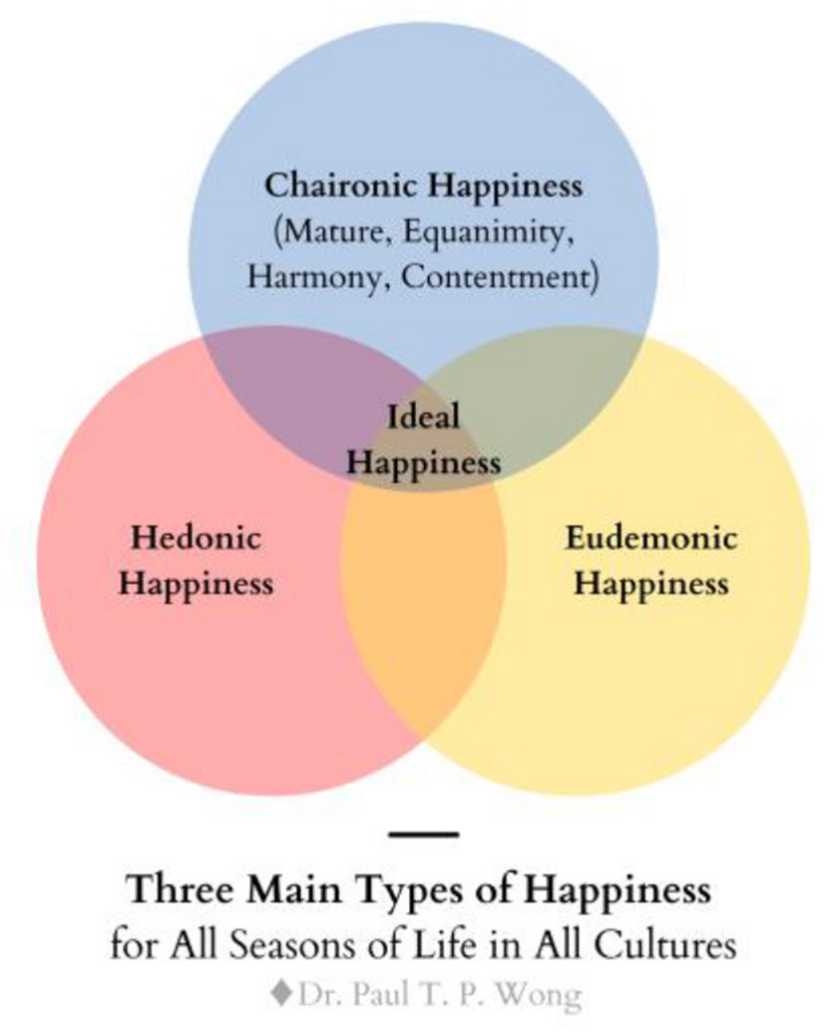
Different types of happiness.

**Figure 2:**
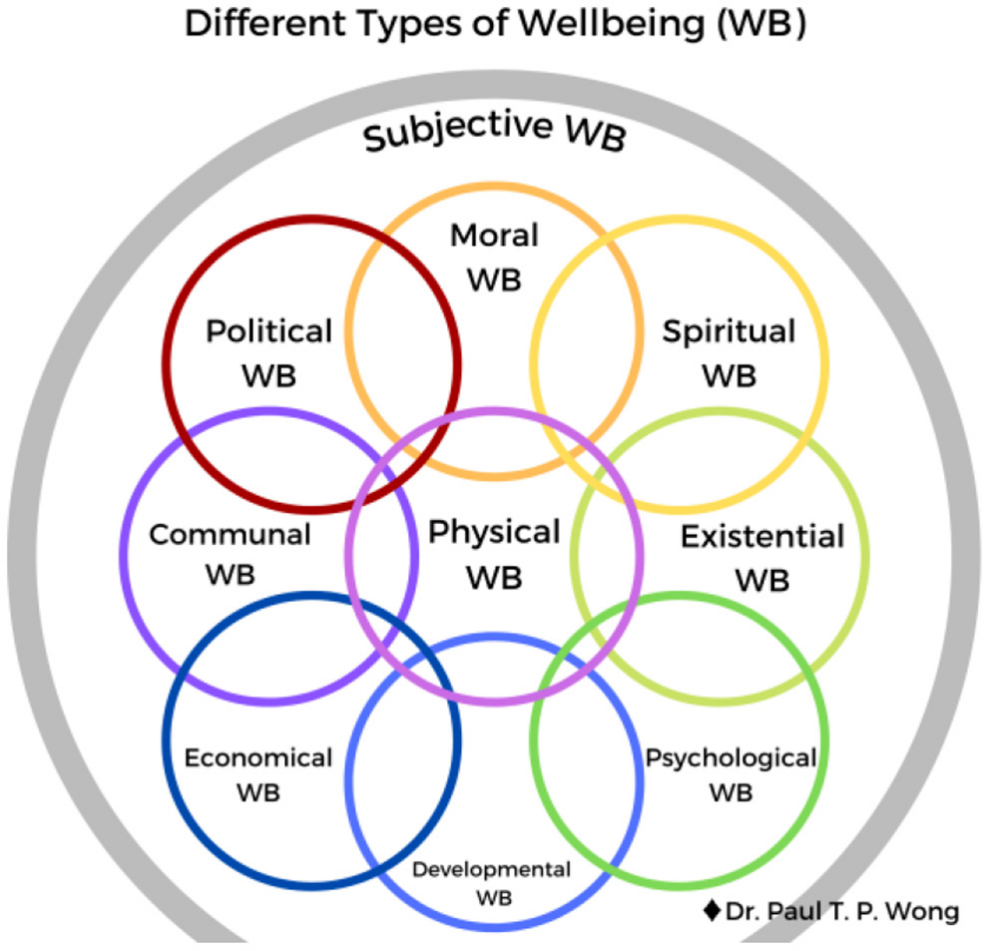
Different types of well-being.

**Figure 3:**
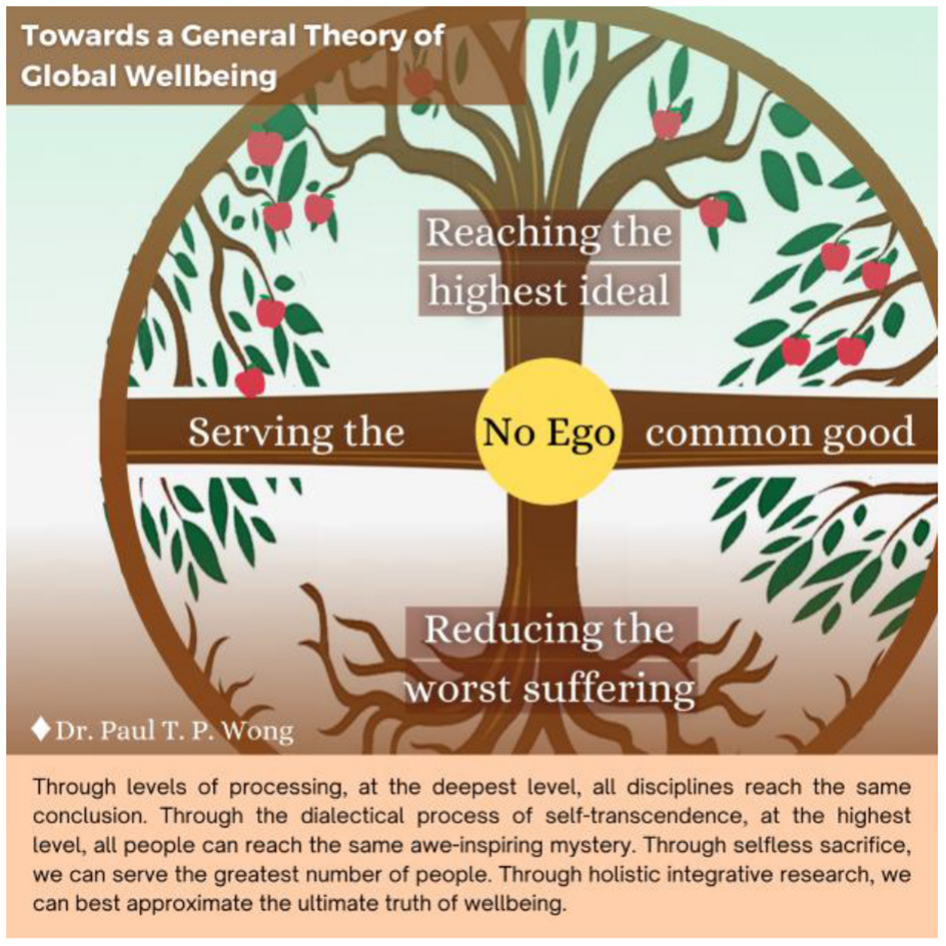
Toward a general theory of global well-being.

See Figure 1 depicting different types of happiness and Figure 2 for different types of well-being. Also, see the tentative global theory of well-being of Wong, which represents the ideal of Taoism (Figure 3).

Ancient philosophies, such as Taoism and Buddhism, also contribute to our understanding of mature happiness. For example, I-Ching offers the Way to live a good life in an uncertain and ever-changing world. Happiness is never just about individual pursuit, but about the way to achieve harmony as reflected by the Taoist concept of the union between Heaven and people (天人合一), or living in harmony with heaven, earth, and people (天地人和).

Therefore, the less-traveled path to happiness is to attain inner peace and harmony with self, others, and Heaven and Earth.

A philosophical understanding of paradoxes and contradictions provides the necessary background for the psychology of mature happiness. Wong and Bowers (2018) made a similar argument that mature happiness, characterized by inner peace and harmony, comes from the wisdom and courage to transcend opposites. More recently, Lomas (2021) declared that: “an overarching definition of well-being is offered: the dynamic attainment of optimal balance and harmony in any–and ideally all–aspects of life.” (Lomas, 2021, p. 5). Thus, in addition to the emotion of awe, mature happiness comes from cultivating wisdom, courage, and virtue in transcending suffering (Wong, 2020a, 2021a). True happiness comes from being the light to banish the darkness in the world. It means the self-transcendental way is enduring the necessary suffering and self-sacrifice for the greater good. According to Wong (2021b), there are three major types of happiness as shown in Figure 1. Mature happiness is basically spiritual (noetic) happiness based on self-transcendence, but it could also be known as charionic (Wong, 2011 PP 2.0), because the Greek word for rejoicing in suffering is Xαíρ*ετε* (chairete) as found in Philippians 4:4 of the Bible.

### Frankl's Self-Transcendence Model

The earliest and most influential theory of self-transcendence was developed by Frankl (1946/1985). Researchers routinely credit Frankl as the father of meaning-focused therapy, but they fail to understand conceptualization of meaning in terms of self-transcendence that Frankl proposes. A deep sense of meaning involves the volition to exercise the will to the meaning of an individual to pursue self-transcendence for the common good regardless of sacrifice and suffering. Meaning is not only anchored on subjective feelings, but also on an objective behavioral commitment to devoting one's life to loving something or someone greater than oneself, such as loving others or serving society according to one's calling. This paradoxical truth of self-fulfillment is clearly stated in this quote:

“Only to the extent that someone is living out this self-transcendence of human existence, is he truly human or does he become his true self. He becomes so, not by concerning himself with his self's actualization, but by forgetting himself and giving himself, overlooking himself and focusing outward” (Frankl, 1977/2011, p. 36).

When probably understood, self-transcendence represents the most beautiful story about what is good and noble about humanity. It is an uplifting story about human beings, individually or collectively, who dedicate and sacrifice themselves in order to lift up others beyond their limitations and sufferings to a higher ground of faith, hope, and love. Thus, in addition to this re-orientation, the willingness to suffer for the common good is another defining characteristic of self-transcendence, because it is not possible to achieve the highest ideal or deepest meaning of an individual without any sacrifice or suffering.

In short, Frankl attempted to restore the soul or the noetic (spiritual dimension) to psychology and society (Wong, 2021c). Thus, the meaning of life or self-transcendence is about developing the gift an individual has so they can give their best to serve the world (Wong, 2016a).

Research supports Frankl's definition of meaning in terms of a search for self-transcendence. For example, Harris et al. (2018) showed that finding meaning is an essential element of self-transcendence. McClintock (2015) reported that self-transcending gratitude may be the very key to how we can become change agents of making a difference in the world. A sense of the self-transcendental motivates individuals to live a meaningful life and use their gifts to make a positive contribution to society (Wong, 2014a, 2016a).

The remembrance of Wong (2021c) on the contribution of Viktor Frankl to self-transcendence revolved around three themes:

1. Self-transcendence is an awe-filled lifestyle of serving a cause passionately.
2. Self-transcendence is the core of meaning-focused therapy and an important adjunct for all therapeutic dimensions.
3. Self-transcendence has become the foundation of existential positive psychology.

According to Frankl (1946/1985), self-transcendence is the essence of human nature; it presents a healthy spiritual core with its yearning to strive toward the sacred and the service of others:

“Only to the extent that someone is living out this self-transcendence of human existence, is he truly human or does he become his true self. He becomes so, not by concerning himself with his self-s actualization, but by forgetting himself and giving himself, overlooking himself and focusing outward.”

Self-transcendence involves a change of mindset from *what I can get from life* to *what I can give to life*. It involves a shift from the horizontal dimension of being preoccupied with worldly success and comfort to the vertical way of living that focuses on the spiritual needs an individual has for meaning, personal growth, and serving something sacred or greater than oneself. This quantum shift is necessary to fill the void of the empty self and enable us to become our best version despite internal and external limitations. This is why Frankl suggested that the best way an individual can discover their own calling is to discover both what gift they can offer and what life demands from their own lives. Thus, we are responsible, both ethically and instrumentally, for the well-being of our neighbors because we are an integral part of a relational world. This is why being ethical and benevolent toward others is an inherent part of self-transcendence (Wong and Reilly, 2017).

### Frankl's Two-Factor Theory of Self-Transcendence

**Figure 4:**
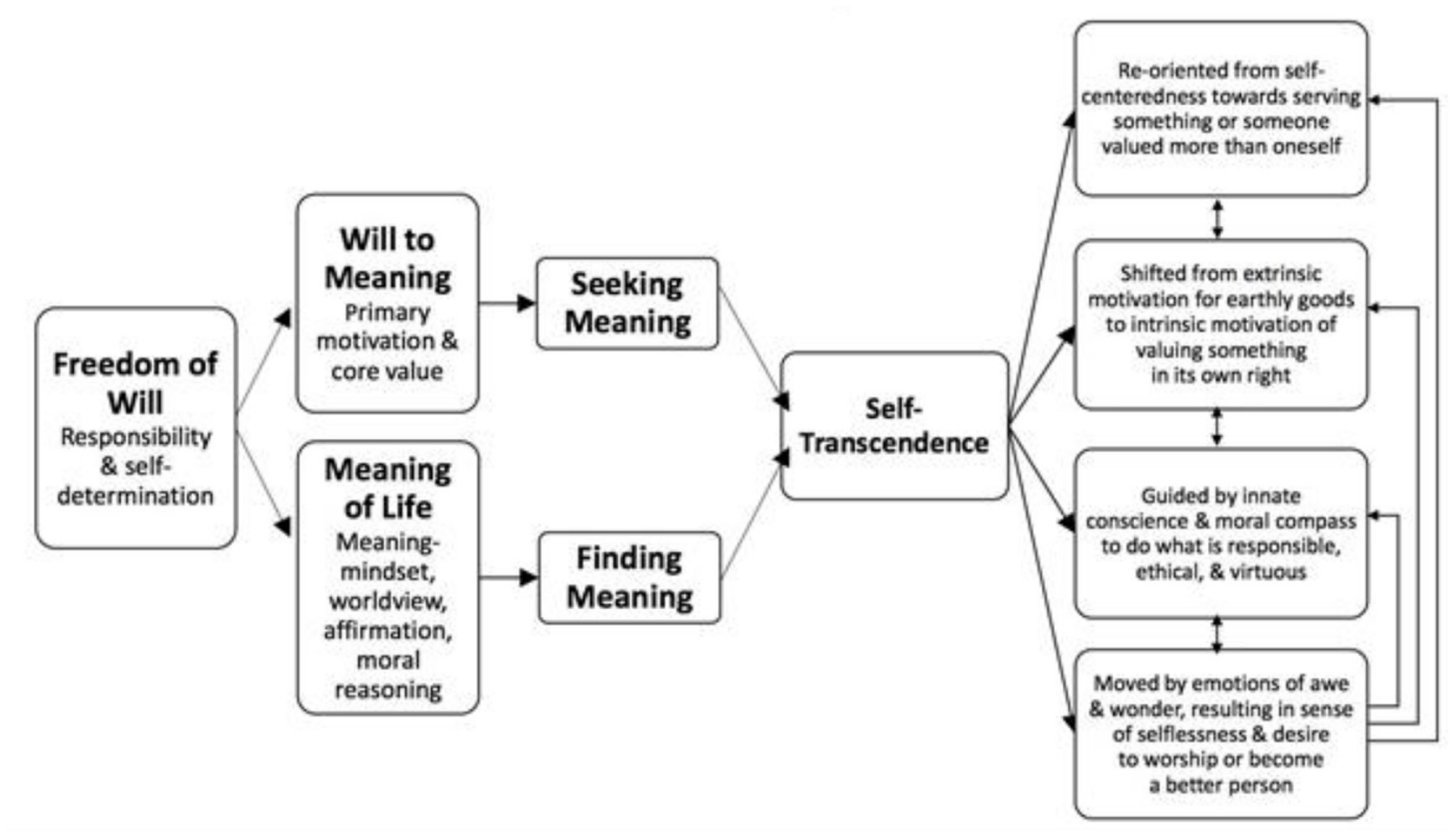
Two-factor theory of self-transcendence proposed by Frankl.

As shown in Figure 4, the theory of self-transcendence proposed by Frankl rests on two factors.

#### Will to Meaning as a Motivational Factor

The will to meaning, the motivational factor that propels people from point “A” toward “B” through the process of self-transcendence, is considered by Frankl as a primary motivation. From the perspective of the terminal values of life, self-transcendence was one of the universal values according to Schwartz (1992, 1994). The will to meaning consists of both the push and pull motivations in our own striving toward a meaningful goal, which is pushed by the yearning of the soul and pulled by a future purpose to be fulfilled.

#### Meaning-Mindset as the Cognitive Factor

The meaning-mindset (MM) consists of the worldviews or assumptions of an individual affirming that life has inherent meaning and value (Wong, 2012). If mindfulness adds clarity by reducing the emotional reactivity of an individual, MM provides the lens that adds content and depth to their perception. It enables people to see things from a deeper spiritual perspective. Furthermore, MM enables one to see the connectivity of all people and all living things and to discover the significance of ordinary events. In short, we tend to discover or see what we value or search.

Contrary to positive psychology's preference to emphasize the positive emotion of awe, the emphasis of Frankl is on will and mindset. The MM facilitates the discovery of meaning and significance and the selection of worthy life goals to pursue. These two factors work together to contribute to the discovery of self-transcendence at all three levels. Meaning-mindset opens our own perceptions to all the hidden meanings in ordinary life experiences, including sunsets to sunrises, the starry skies and snow-capped mountains, the spontaneous play of little children, and the wrinkled smiling faces of old people; these experiences can move us with a sense of wonder and appreciation of life.

The importance of having a realistic and yet positive worldview is emphasized (van Deurzen, 2014; Wong, 2021d). Research also indicated that the worldview of an individual that life has inherent meaning that facilitates goal-striving toward sacred emotions (Emmons, 2005) or eudaimonic happiness (Braaten and Huta, 2016). In addition, Haidt (2000, 2003) provided evidence that being open to the possibilities of discovering meaning and connecting with something larger increases the likelihood of awe and the positive emotion of elation. Together, these two factors contribute to the pursuit and discovery of self-transcendence, which has four defining characteristics.

### The Four Defining Characteristics of Self-Transcendence

#### A Shift in Focus From the Self to Others

This involves a re-orientation from egotistic concerns or selfishness toward something greater or someone more valued than oneself. There is a new sense of selflessness in being connected with something vast or grand or someone more important than our own lives. While the will to meaning provides the necessary motivation to actively engage in searching for self-transcendence, the MM provides the worldview and openness to discover something that transcends selfishness and personal limitations. This re-orientation may happen at three levels: the transpersonal, life as a whole, and situational. At the transpersonal level, there is a perceived connection with God, nature, or all living things. There is a new openness to the spiritual/transcendental realm and the possibilities of meaning, beauty, and goodness all around us, which facilitate our discovery of all these ideals. At the level of life as a whole, there is an awareness of one's worthy life purpose or the unconditional self-acceptance and affirmation that their lives have inherent meaning and value, independent of their possessions or achievements. An awareness of major life transitions and the mortality an individual has can shift the focus from a preoccupation with earthly concerns to the larger schemes of things and the transcendental realm. Spiritual conversion may also shift the focus from the city of earth to the city of heaven (Tolstoy, 1882/1921; Augustine, 2009).

#### A Shift in Values

This involves a shift in emphasis from extrinsic motivation to intrinsic motivation. Intrinsic motivation refers to doing something in its own right, such as playing, doing good deeds, or caring for friends and loved ones. Such activities are not only inherently rewarding and worthwhile but also beneficial to others and make the world a better place. One engages in self-transcendence for its own sake, rather than as an instrument to serve some other extrinsic motive. It also entails the willingness to sacrifice self-interest for serving the greater good or a higher purpose. Intrinsic motivation also means pursuing some goal that has intrinsic or inherent value, which is the value that is good in itself and for its own sake, such as the goal of helping others, saving lives, or saving planet earth. The study of Frankena (1973) regarding the list of intrinsic values includes life, consciousness, love, virtue, justice, and all things that are morally good inherently. These shifts in focus and values may result from a personal encounter with a life-changing event, such as surviving 9/11 or a serious illness, but also from a shift in perspective from a success orientation to a spiritual or meaning orientation as a result of reading the books of Frankl or seeing a meaning therapist. These shifts emphasize that all people are hardwired for connecting with each other and with a higher power or the cosmos. Therefore, relational pursuits motivated by love and compassion are deeply satisfying to the extent they meet the deepest spiritual needs of an individual.

#### An Increase in Moral Concern

There is increased attention to the moral dimension in the pursuit and action of a goal by an individual. The above shifts are aligned with the moral compass of an individual, which includes their own innate conscience (natural moral law), religiously or theologically based moral law, and some culturally based normative vision of moral values and virtues. The cardinal virtues according to Plato and Confucius, or the normative ethics of the golden rule, are part of the moral compass that differentiates true self-transcendence from pseudo- self-transcendence, such as terrorist suicide bombers. In addition, we are endowed with the capacity for moral reasoning. We evaluate some goals and actions as moral when they are consistent with our moral compass. Moral responsibility is important to ensure that we are sensitive to the well-being and rights of other people when we strive for our goals and make decisions.

#### Emotions of Awe

All the above three characteristics may trigger emotions of elevation. These emotions include awe, ecstasy, or amazement because of something extraordinary in its vastness (God or nature), ability (exceptional accomplishment), or goodness (special kindness of one person toward another). This feeling of awe is part of a life-transforming experience because it moves people toward worship or becoming better and more responsible individuals.

Peak experiences (Maslow, 1964) and the experience of timelessness inflow (Csikszentmihalyi and Hunter, 2003) often involve the emotion of awe. Watching sunsets, listening to classical music, or worshiping God in a church or temple can all contribute to the emotion of awe. Dobson (2015) provided a thorough review of the literature on awe and concluded that awe is related to a diminished sense of the self.

In a qualitative study, Bonner and Friedman (2011) also identified similar themes in their analysis of the accounts of participants when they experienced awe. Specifically, they found that when participants experienced awe, they described that they were part of something larger than themselves. In addition, the experience of awe was associated with a decreased attention to the self. Generally, the emotion of awe contributes to a shift in focus, well-being (Rudd et al., 2012), and self-transcendence goals (Seaton and Beaumont, 2015). Furthermore, Kristjánsson (2015) has also examined how awe is related to humility and virtue.

In view of this finer differentiation of the four defining characteristics, Wong developed the 24-item Self-Transcendence Measure (STM) with six items pertaining to each dimension, which was later revised into 21-items after eliminating items with double-loadings. This revised STM has already been used in a few studies to demonstrate its validity. For example, the study by Dhillon (2020) on path analysis has empirically supported the self-transcendence spiral hypothesis of Wong (2017), which posited that, when there is a clear shift in focus from the self to the other as measured by STM, there is an increase in the “meaning-virtues-happiness” cycle. In another study, Dhillon (2021) showed that STM is negatively associated with depression, but positively correlated with social support, meaning in life, and positive traumatic growth in divorced women. Finally, the study of Dhillon and Singh (2021) reported that, in young adults, humanity was the only virtue correlated with STM, whereas in the middle-aged sample, all virtues as measured by the Values in Action (VIA) Survey of Peterson and Seligman (2004) were correlated with STM.

### Wong's Self-Transcendence Paradigm

**Figure 5:**
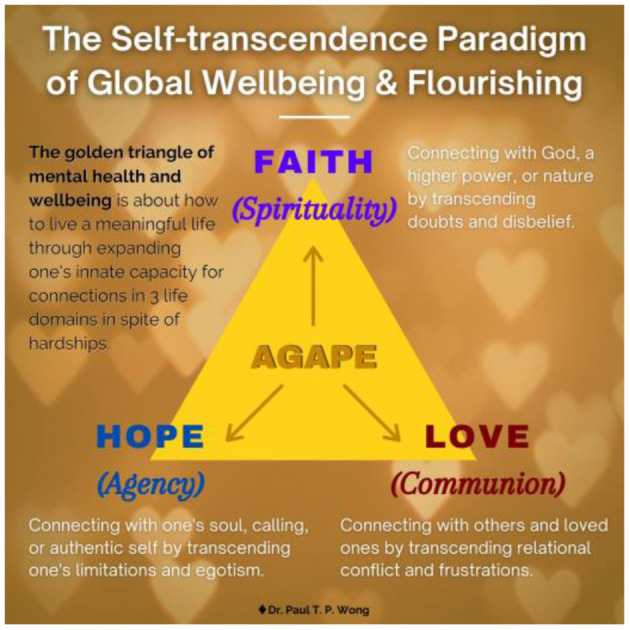
The golden triangle of self-transcendence.

According to Wong's self-transcendence paradigm (Wong, 2021a), self-transcendence can only be experienced through satisfying the deepest yearning of the soul for connections with our own selves, with others, and with God, which are the three major life domains, as shown in Figure 5.

Frankl (1946/1985) got it right when he said that love is the ultimate truth: “The truth-that love is the ultimate and highest goal to which man can aspire.” Indeed, the most promising way to live a good life is to aim for the highest ideal of unconditional love (agape) for the self, for others, and for God as shown in the golden triangle.

This requires a radical reorientation from egotistic concerns to striving for the ideal of becoming the best in order to serve others and glorify the source of life and all blessings. Such a striving toward the ultimate concern or the highest good will lead to an adaptive balance between all opposing forces and result in inner peace and harmony even during COVID-19.

The concept of faith, hope, and love can be grasped intuitively because we all have the innate capacity to believe, hope, and love. Furthermore, this is because we all have experienced these human qualities even when we are not fully aware of them. For example, whenever we pledge to marry someone or enter into a business relationship with someone, we are taking a leap of faith because people are complex, and we can never fully know or understand that our partner will be trustworthy or compatible in the future. Similarly, when we are in desperate situations beyond human control, we naturally pray to God or a higher power for help (Goodman, 2020). To protect ourselves against the terror of death, we may unconsciously identify with our cultural beliefs, according to terror management theory (Pyszczynski et al., 2004).

Likewise, hope is also an optimistic state of positive expectation, but it is often related to having confidence in our own efficacy. Whatever our goal or plan, we are motivated to work toward such only when we have some agentic hope of success. According to hope theory (Snyder and Lopez, 2009), hope is defined as the perceived ability to discover the pathways to desired goals and the efficacy to achieve these goals. There is vast literature supporting hope theory. While faith makes all things possible, hope makes the prospect of our future plans brighter, and love connects us all together.

When it comes to love, compassion, and relationships, the literature is beyond measure. Supreme human achievements, from medicine and science to religion and literature, are motivated by love. Some of the best poems and pieces of music are inspired by love. There is a very long list of publications on the importance of loving and connecting with others (for examples, see Adler, 1938/2011; Fromm, 1956/2019; Ainsworth, 1989; Baumeister and Leary, 1995). There is a consensus that humans are hardwired to connect (Golemen, 2007; Siegal, 2010).

At the personal level, we desire to love and to be loved. Intuitively, we believe that love is the antidote to loneliness and the key to happiness. A life without love is like a garden without water. We need love in order to satisfy the deepest yearnings of our souls and make our lives complete.

In sum, our souls need faith, hope, and love just as our bodies needs air, food, and water. When any of these basic psychological/spiritual needs are not met, we will experience a loss of life balance and an increase in distress. In this study, we tested this hypothesis (see the main study) and found that the evidence supports our claim that these three pillars of mental health (or the golden triangle of well-being) are capable of not only enabling us to cope effectively with the suffering of COVID-19, but also transforming us into better and stronger human beings.

**Figure 6:**
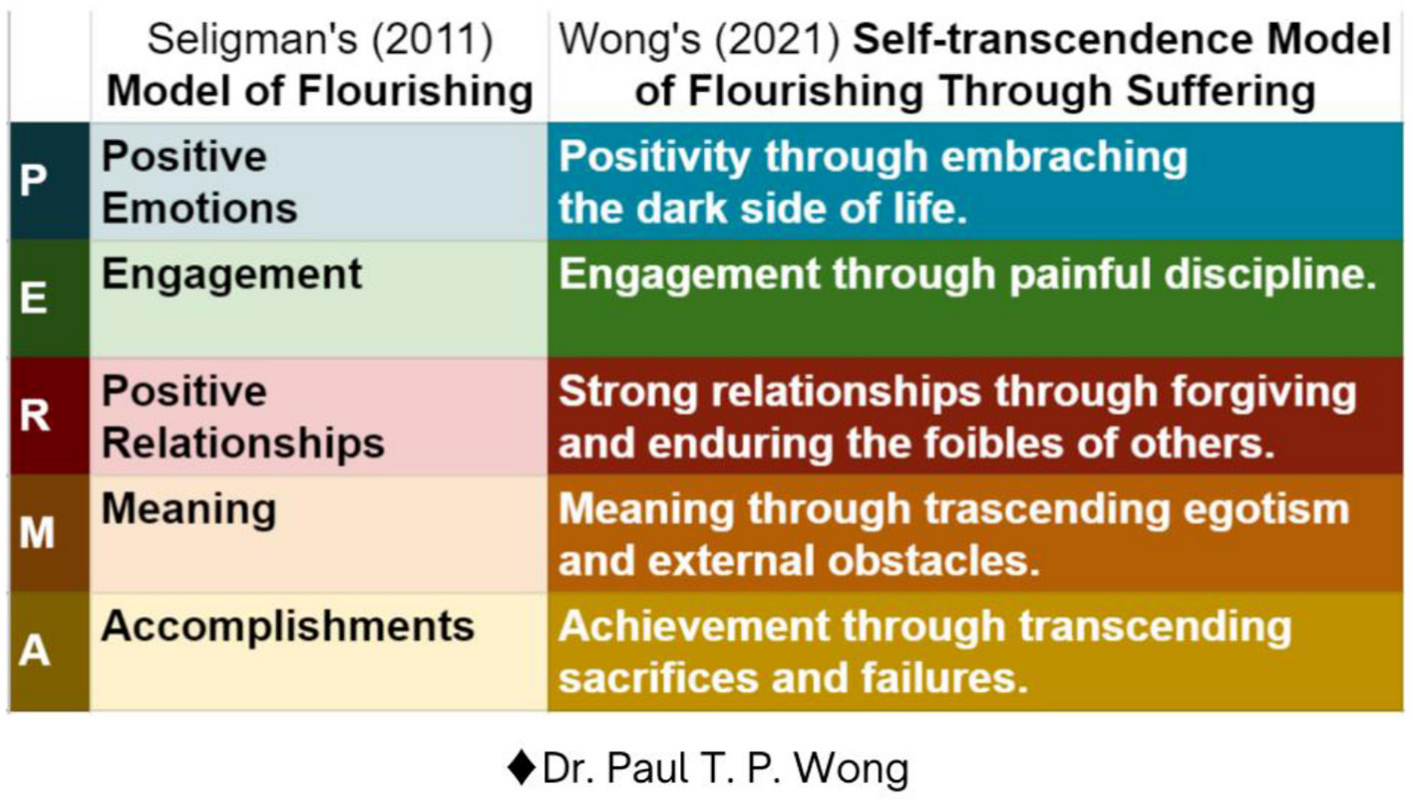
Seligman's (2011) model of flourishing compared with Wong's (2021a) self-transcendence model of flourishing through suffering.

Wong (2021a,d) proposed that the self-transcendence approach to global well-being involves so many fundamental changes in assumptions and methodologies that it represents a new paradigm of flourishing through suffering. It hypothesized that all the good things in life are on the other side of fear, and all the best things in life are on the other side of suffering. Therefore, it is difficult, if not impossible, to achieve flourishing without going through the gates of overcoming adversity as reflected by Figure 6.

Figure 6 indicates that only by incorporating the realism of transcending or overcoming the harsh reality of life can we achieve flourishing, especially in the era of COVID-19, when suffering can no longer be ignored or avoided. Thus, it requires a radically different set of basic assumptions for well-being:

1. Everything exists in polarity. The old way of binary, either-or thinking is that everything is either positive or negative as two opposite poles on the same dimension. The new way is to see positive and negative as two separate but complementary dimensions, thus allowing maximum cognitive flexibility and emotional agility in coping with the world.
2. The old way is to choose between one of two opposite poles. The new way is to have a mind big enough to hold two opposing ideas or a heart big enough to hold two opposing emotions. This involves creative tension and creative thinking to transcend the two opposites.
3. The old way is to seek to maximize the positive and minimize the negative, to accept one position and reject the opposite one. This will result in a polarized mind and a divided society. The new way is to navigate an optimal balance between two opposites to maintain dynamic harmony and balance as the core of well-being.

In sum, self-transcendence is the less-traveled road toward personal growth and well-being. This narrow path will never be as popular as the broad way to happiness because it involves tough choices in favor of what is true, good, and beautiful, but it will lead you to live a life of meaning and fulfillment.

In the age of COVID-19, when people all over the world are overwhelmed by loneliness, anxiety, and stress, the practice of self-transcendence will lead to a more compassionate and harmonious society (Wong, 2016a) and a return to the virtue of humility and selflessness love. In short, the self-transcendence paradigm requires a very different way of doing research and intervention to promote global well-being.

### Development of the STM

The STM was designed as a psychological instrument to measure self-transcendence. The 24-items of the scale (see the Appendix) were generated by Wong primarily based on the four-factor model concept of self-transcendence described earlier. The face validity of these items was first screened by a group of 30 graduate students taking a course on self-transcendence and by three psychology professors (co-authors of this scale at the University of East London). The list was then presented to a group of 30 scholars (including philosophers, theologians, and psychologists) supported by a Templeton Grant on self-transcendence (Guerra, 2017) for their feedback. Some of the 24 statements were reworded as a result of their feedback as shown in the Appendix.

### Factor Structure of the STM

#### Study 1

To examine the factorial structure of the STM, a series of studies was conducted. Participants were recruited using Mechanical Turk (www.mturk.com) (Amazon Web Services (AWS), Seattle, Washington), which is a web portal that facilitates paying participants to complete tasks such as surveys. Recruiting participants from Mechanical Turk has exhibited comparable research results as more conventional methods, with diverse participants and the sample comprising good generalizability (for example, see Buhrmester et al., 2011). Furthermore, it avoids the problem of using weird samples with a more representative sample (Rad et al., 2018). Participants in these studies were paid varying amounts ranging from $0.4 to $1.50 based on the length of the questionnaire in the study.

An initial exploratory factor analysis was conducted on the 24-item measure using data collected from 240 participants (results from five participants were excluded because they did not answer a control question correctly). Among the 235 remaining participants, 138 (58.7%) were men, 96 (40.9%) were women, and 1 (0.4%) was other. Their ages ranged from 19 to 73 years (*M* = 33.76; *SD* = 10.74), and 193 (82.1%) reported being from USA, 34 (14.5%) from India, and 8 (3.4%) from other countries. All reported having English as their first language. A maximum likelihood factor analysis using Promax rotation (IBM, Armonk, NY) was conducted and resulted in two factors being retained. This two-factor solution accounted for 43.3% of the variance, with the first factor explaining 37.9%. However, this analysis did not yield a clear factor structure. The highest loadings on the first factor were items related to meaning. Several moral items had the highest factor loadings on the second factor and some items had dual loadings or low loadings on both factors.

#### Study 2

The scale items were reduced to 18 of the strongest loading items, and a second exploratory factor analysis was conducted on the results from 248 participants (5 were removed due to not answering the control question correctly) who completed this scale version. Among the 248 remaining participants, 175 (70.6%) were men and 73 (29.4%) were women. Their ages ranged from 19 to 65 (*M* = 31.71; *SD* = 8.3) years old, and 96 (38.7%) were from USA, 134 (54%) from India, and 18 (7.3%) from other countries. English was reported as the first language by 217 (87.5%) participants. A maximum likelihood factor analysis of these 18 items using Promax rotation resulted in two factors being retained. This two-factor solution accounted for 43.1% of the variance, with the first factor explaining 37.3%.

#### Study 3

The scale was further trimmed by eliminating an additional six of the original items to produce a 12-item scale that was completed by another 255 participants; 20 were excluded for not answering the control question correctly. Among the 235 remaining participants, 134 (57%) were men and 101 (43%) were women; their ages ranged from 18 to 72 (*M* = 34.94; *SD* = 10.31) years old. Among the participants, 166 (70.6%) were from the USA, 51 (21.7%) from India, and 18 (7.7%) from other countries. For 222 (94.5%) participants, English was listed as their first language. A maximum likelihood factor analysis (Promax rotation) was employed to retain two factors. This two-factor solution accounted for 42.8% of the variance, with the first factor explaining 35.5%. Two items loaded on both factors and were subsequently removed to produce the final 10-item Self-Transcendence Measure-Brief (STM-B).

### Psychometric Properties of the STM-B

#### Study 4

To examine the construct and concurrent validity of the STM-B, 254 participants were recruited (34 were subsequently excluded from the analyses due to not answering the control question correctly) to complete the scale along with the measures noted below. Among the remaining 220 participants, 154 (70%) were men, 65 (29.5%) were women, and 1 (0.5%) was other. Their ages ranged from 20 to 64 (*M* = 31.37; *SD* = 8.34) years; 90 (40.9%) were from the USA, 107 (48.6%) from India, and 23 (10.5%) from other countries. Additionally, 202 reported speaking English as their first language.

In addition to the STM-B, the following measures were completed: The self-transcendence subscale from the Sources of Meaning Profile-Revised (Reker, 1995), the Collectivism subscale from Sources of Meaning Profile-Revised (Reker, 1995), and the Benevolence and Universalism subscales from the Schwartz's Value Survey (Schwartz, 1992). The STM-B (M = 29.87; *SD* = 6.58; *N* = 220) was found to have very good internal consistency (Cronbach's alpha = 0.88).

**Figure 7:**
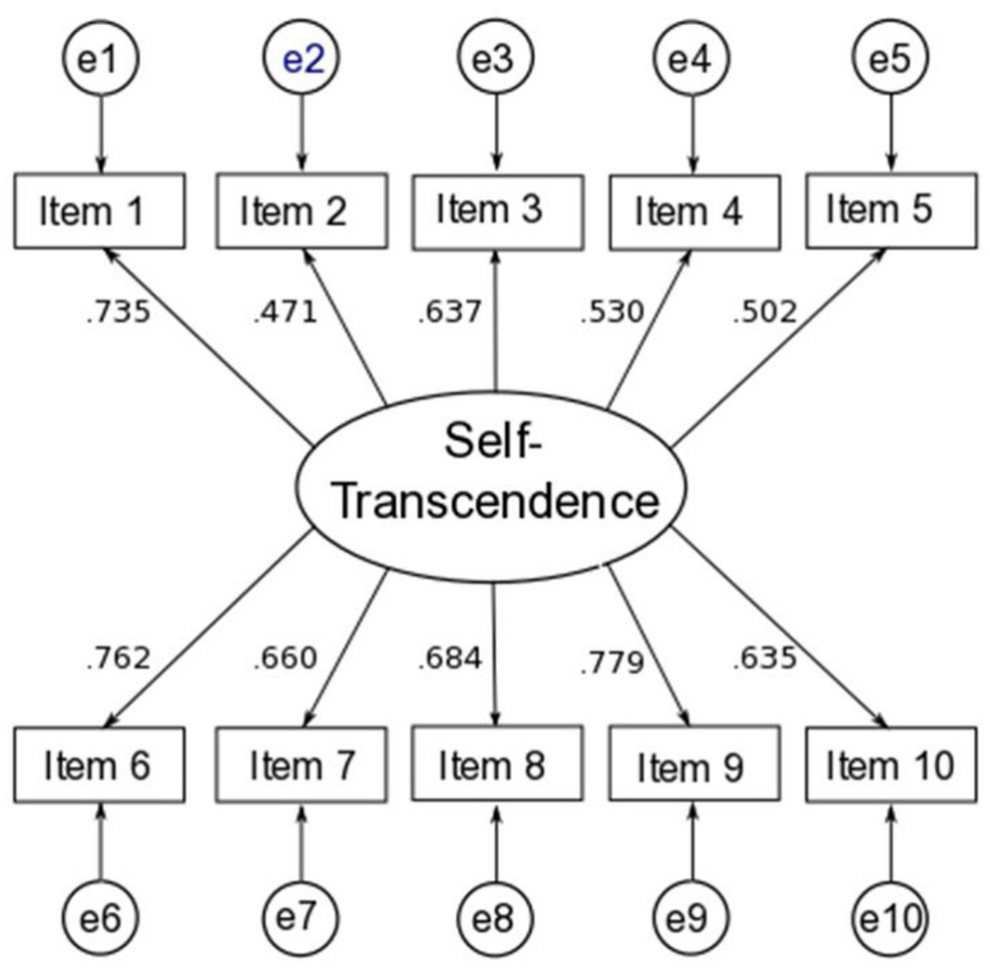
Standardized regression weights for the one-factor model of self-transcendence (*N* = 220). The models were identified by setting the factor loading of the first indicator of the latent construct to 1. The presented values are standardized by setting the variances of both the observed and latent variables to unity.

A confirmatory factor analysis (CFA) was carried out using the Lavaan package (R Foundation, Vienna, Austria) (Rosseel, 2012) in R (R Core Team, 2016) to verify that all STM-B items were indicators of a single latent variable. As the item responses were not normally distributed, the robust estimator maximum likelihood method was used; the results of this analysis indicated that a single factor model provided a good fit to the data (χ^2^ = 45.062, *p* = 0.119, *N* = 220; CFI = 0.978; RMSEA = 0.036). The results of the CFA are summarized in Figure 7.

#### Study 5

To further examine the psychometric properties of the STM-B, including test-retest reliability, an additional study was conducted that involved participants completing a package of scales at two points in time.

**Time 1**. At Time 1, data were collected from 252 participants. There were 29 participants who failed to answer the control questions correctly and were removed from the study. Among the remaining 223 participants, 123 (55.2%) were men and 100 (44.8%) were women. Their ages ranged from 18 to 68 years (*M* = 35.4; *SD* = 11.44), and 159 (71.3%) were from the USA, 40 (17.9%) from India, and the remaining 24 (10.8%) from other countries. Additionally, 211 (94.6%) reported English as their first language.

In addition to the STM-B, scales included at Time 1 were nine measures of various aspects of well-being: the Mature Happiness Scale (Wong and Bowers, 2018; 12 items), the Satisfaction with Life Scale (Diener et al., 1985; 5 items), the Harmony in Life Scale (Kjell et al., 2016; 5 items), the Positive and Negative Affect Scale (Watson et al., 1988; 20 items), the Questionnaire for Eudaimonic Well-being (Waterman et al., 2010; 21 items), the Peace of Mind Scale (Lee et al., 2013; 7 items), the Objective Life Condition Assessment (Wong, 2018; 1 item), and the Single Item Narcissism Scale (Konrath et al., 2014; 1 item).

The coefficient alpha for the STM-B was 0.87, indicating that the internal consistency was very good in this sample and comparable with our previous findings. Table 1 shows the means and SDs for each of the measures, as well as the correlation between STM-B and each of the other Time 1 measures. The STM-B had significantly moderate to moderate-high correlations with most of the measures of well-being. Interestingly, the STM-B had virtually no correlation with the measure of negative affect (PANAS negative scale) or the measure of narcissism (Single Item Narcissism Scale).

**Table 1:** Means, standard deviations, and Pearson correlations between STM-B and measures of well-being (*N* = 220).

	***M***	***SD***	**1**	**2**	**3**	**4**	**5**
1. Self-Transcendence Scale-Brief	29.9	6.58	–				
2. Sources of Meaning Profile Revised-ST^a^	27.7	4.83	0.76	-			
3. Sources of Meaning Profile Revised-CS^a^	21.5	3.98	0.72	0.76	–		
4. Schwartz Value Survey-Benevolence^b^	29.5	7.29	0.38	0.35	0.23	–	
5. Schwartz Value Survey-Universalism^b^	45.9	11.09	0.34	0.33	0.23	0.79	–

*All correlations displayed are significant at the p < 0.01 level*.

a *Reker (1995) and*

b *Schwartz (1992)*.

**Time 2**. Among the 223 Time 1 participants, 140 (62.7%) successfully completed the Time 2 questionnaire. Among these Time 2 participants, 75 (53.6%) were men and 65 (46.4%) were women. Their ages ranged from 18 to 65 years (*M* = 37.2; *SD* = 11.28), and 106 (75.7%) were from the USA, 23 (16.4%) from India, and the remaining 11 (7.9%) from other countries. Additionally, 137 (97.9%) reported English as their first language.

The Time 2 questionnaire included the following measures also administered at Time 1: the STM-B, the Mature Happiness Scale, the Satisfaction with Life Scale, and the Harmony in Life Scale (Kjell et al., 2016; 5 items), and the 15-item Self-Transcendence Scale (Reed, 1986).

The test-retest reliability for the STM-B was high (*r* = 0.7, *p* < 0.001) over 3–4 weeks. The internal consistency of STM-B at Time 2 was also high (Cronbach alpha = 0.88), consistent with our previous results.

The descriptive statistics for Time 2 measures are shown in Table 2. The correlations between the STM-B and the three measures of well-being also administered at Time 1 were moderately high and similar to the Time 1 values. Also, the correlation between the STM-B and the Self Transcendence Scale of Reed was moderately high, providing further support for the convergent validity of the STM-B.

**Table 2:** Means, SDs, and Pearson correlation coefficients of the STM-B and measures of well-being at Time 1 (*N* = 223) and Time 2 (*N* = 140).

	**Time 1**			**Time 2**		
	***M***	***SD***	***r***	***M***	***SD***	***r***
1. STM-B	27.81	6.88		28.32	6.98	
2. HILS	25.85	5.98	0.54^***^	25.51	6.30	0.56^***^
3. SWLS	23.57	7.29	0.47^***^	23.26	7.59	0.52^***^
4. M-HAPP	41.19	9.16	0.49^***^	41.35	9.34	0.56^***^
5. PANAS-Pos	33.02	9.13	0.56^***^			
6. PANAS-Neg	17.86	9.75	−0.08			
7. EWB	55.21	11.71	0.68^***^			
8. POMS	24.01	5.98	0.45^***^			
9. R-STS				31.39	8.07	0.65^***^

*** *p < 0.001. M and SD are used to represent mean and standard deviation, respectively*.

*STM-B, Self Transcendence Measure-Brief*.

*HILS, Harmony in Life Scale (Kjell et al., 2016)*.

*SWLS, Satisfaction with Life Scale (Diener et al., 1985)*.

*M-HAPP, Mature Happiness Scale (Wong, 2018)*.

*PANAS-Pos, Positive and Negative Affect Scale - Positive Items (Watson et al., 1988)*.

*PANAS-Neg, Positive and Negative Affect Scale - Negative Items*.

*EWB, Questionnaire for Eudaimonic Well-being (Waterman et al., 2010)*.

*POMS, Peace of Mind Scale (Lee et al., 2013)*.

*R-STS, Self Transcendence Scale (Reed, 1986)*.

### The Main Study: Self-Transcendence as a Buffer on COVID-19 Suffering

#### The Purpose of the Present Study

**Figure 8:**
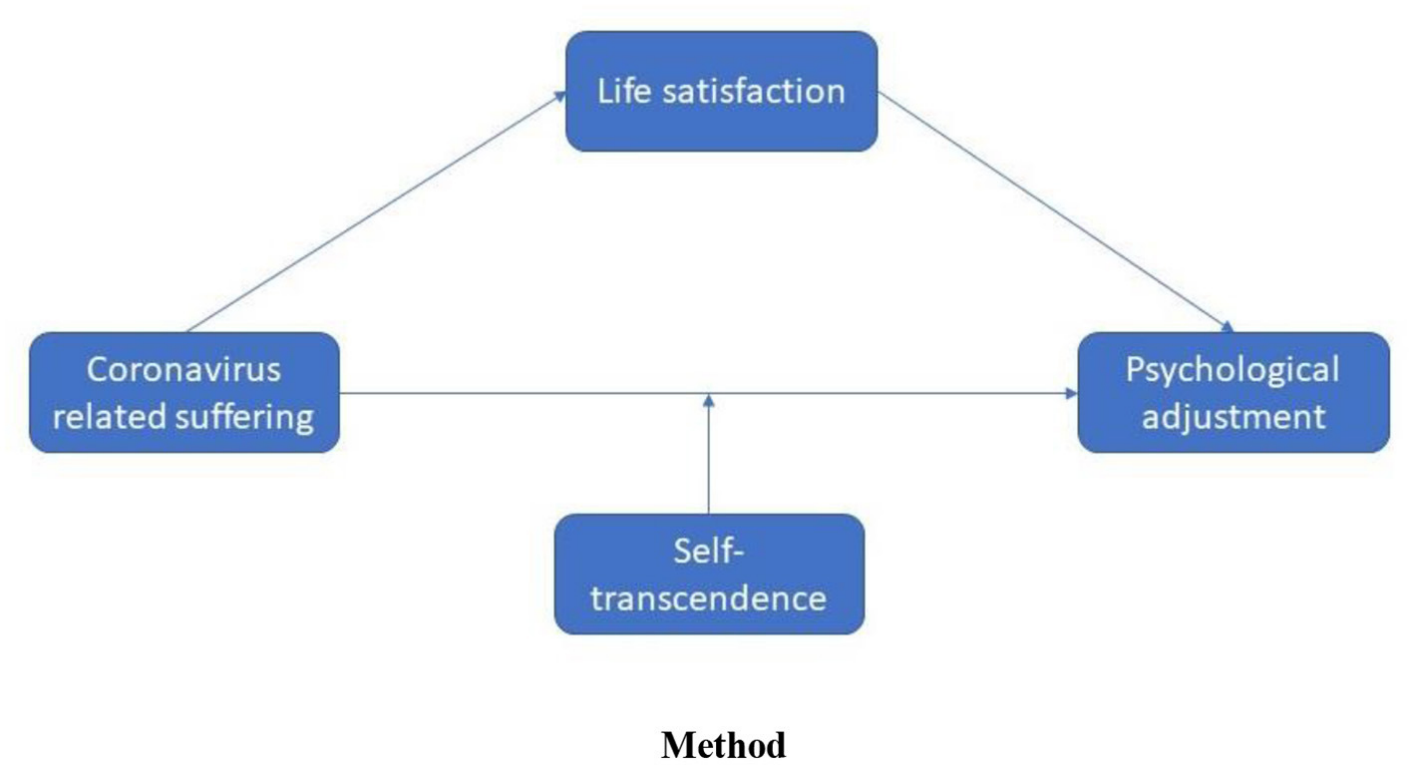
Moderated mediation model demonstrating the association between the variables of the study.

The age of COVID-19 calls for a different approach toward global well-being based on overcoming suffering as advocated by existential positive psychology. In the present study, we primarily explained what self-transcendence is and why it represents the most promising path for human beings to flourish through overcoming and transforming suffering in a complex and uncertain world. After reviewing the literature of a variety of self-transcendence experiences, we first examined the psychometric properties of the development of the STM in the previous five studies. We then tested a moderated mediation model to explore whether life satisfaction mitigated the negative effect of coronavirus suffering on the psychological adjustment of people and whether self-transcendence moderated the mediating effect of it in this association by serving as a buffer against coronavirus experiences (see Figure 8).

## Methods

### Participants

Employing a convenience sample and an online survey, the survey was applied to 183 adults, ranging in age between 20 and 84 years (*M* = 47.55; *SD* = 13.11). The study comprised 62% women and 38% men. According to Kline (2015), an adequate sample size should be 5 or 10 times the amount of the parameters in the path and factor analysis, and a reasonable sample size is about 150 (Muthén and Muthën, 2002; Fritz and Mackinnon, 2007). The sample of this study is thus considered adequate for the analyses. The participants were recruited through social media and email within the affiliated networks of the research team. A web-based survey was created using the study measures and demographic questions. Participants were informed of the voluntary nature of the study and were assured that no identifiable personal information would be collected to ensure anonymity.

### Measures

#### Suffering During COVID-19

The Suffering Measure During COVID-19 (SM-COVID-19) was used to measure the suffering experiences of people during COVID-19, including their adversities (Wong, 2020b). The scale is a 10-item self-report scale, e.g., “Poor physical health condition,” and all items were scored using a five-point Likert type scale, ranging from 1 (not at all) to 5 (a great deal). The internal reliability estimate of the scale with the present study was strong, see Table 3.

**Table 3:** Descriptive statistics and correlations for the study variables.

	***Mean***	***SD***	**Skew**.	**Kurt**.		**1**.	**2**.	**3**.	**4**.
1. Coronavirus suffering	22.62	7.33	0.41	−0.40	0.83	–	−0.01	−0.22^**^	0.50^**^
2. Self-transcendence	33.56	4.83	−0.82	0.59	0.84		–	0.33^**^	−0.20^**^
3. Life satisfaction	24.15	6.29	−0.62	−0.04	0.89			–	−0.47^**^
4. Psychological adjustment	18.22	9.39	0.58	−0.77	0.93				–

** *Correlation is significant at the 0.001 level (two-tailed)*.

#### Personal Meaning

The brief version of the Personal Meaning Profile (PMP-B) was used to assess the perception of an individual of personal meaning and the sense of purpose and personal significance in their lives (McDonald et al., 2012). The PMP-B is a 21-item self-report measure, e.g., I believe I can make a difference in the world. Participants indicated their agreement with each item on a seven-point Likert type scale, ranging from 1 (not at all) to 7 (a great deal). The subscales of the PMP-B included achievement, relationship, religion, self-transcendence, self-acceptance, intimacy, and fair treatment. The subscale scores can be summed to form a total score. The internal reliability estimate of the scale with the present study was strong, see Table 3.

#### Psychological Adjustment

The Brief Adjustment Scale-6 (BASE) was used to assess psychological adjustment problems. It is a six-item self-report instrument, e.g., “I nearly always feel awake and alert,” scoring on a seven-point scale ranging from 1 (not at all) to 5 (extremely) (Cruz et al., 2020). The internal reliability estimate of the scale with the present study was strong, see Table 3.

#### Life Satisfaction

The Satisfaction with Life Scale (SWLS) was used to assess the cognitive emulations of the lives of people. The scale is a five-item self-report scale, e.g., “I am satisfied with my life,” responding based on a seven-point Likert type scale from 1 (strongly disagree) to 7 (strongly agree) (Diener et al., 1985). The internal reliability estimate with this study sample was strong, see Table 3.

### Data Analyses

**Figure 9:**
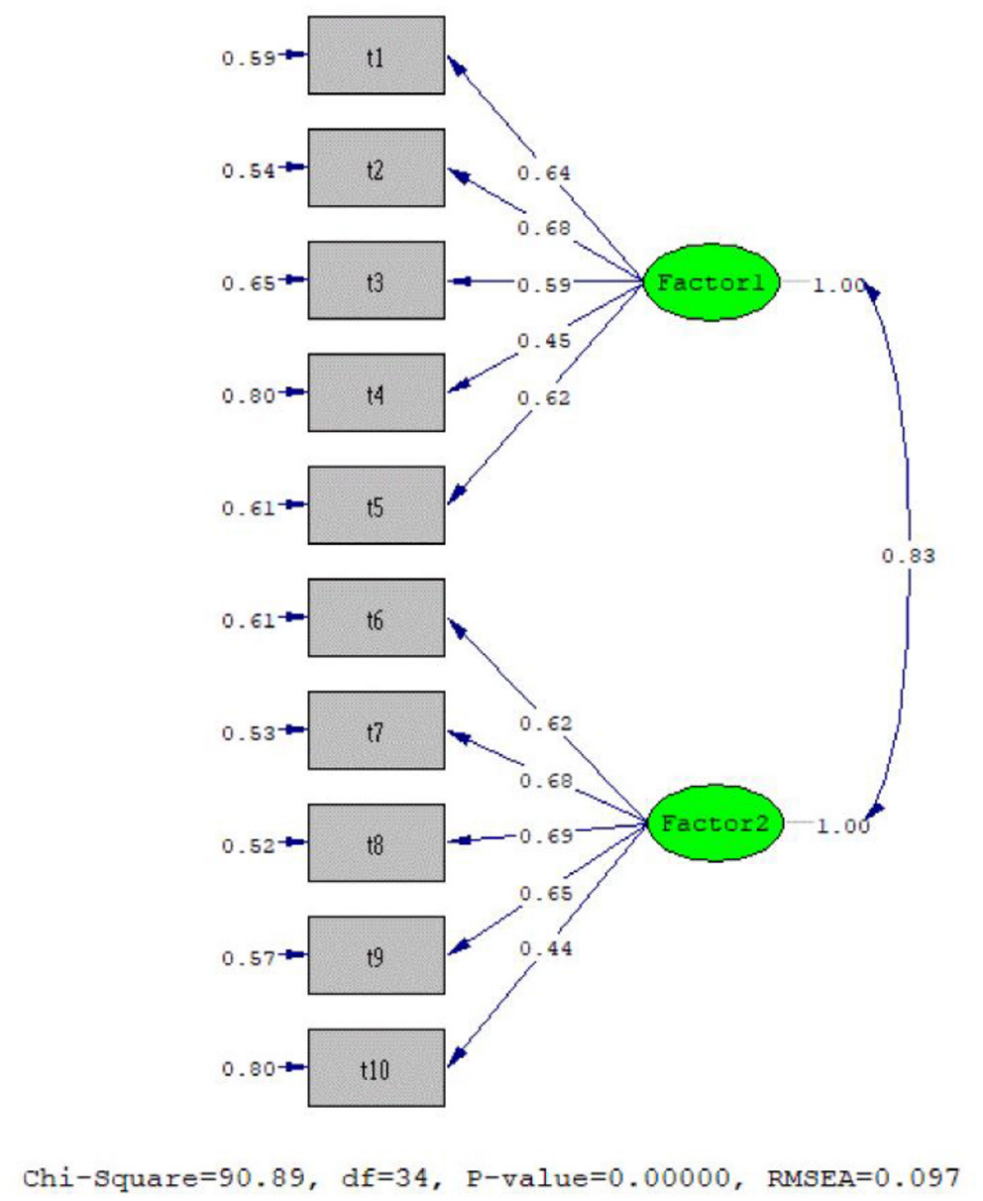
Confirmatory factor analysis results.

The data analyses were performed in two steps. Prior to testing the proposed model, we first examined the psychometric properties of the STM in the present study. The factor structure of the scale was identified using exploratory factor analysis with the sample derived from the previous study [*n* = 213 (69% men, age range = 19–65; *M* = 31.9 and *SD* = 8.52); see in the introduction]. Factor loading scores (λ) ≥ 0.4 are recommended for selecting the items of the measure (Stevens, 2009; Tabachnick and Fidell, 2013). After exploring the factor structure of the measure, we conducted a confirmatory factor analysis to affirm the latent factor structure of the measure with a sample of the present study (Figure 9). The results from this analysis were interpreted using several model fit statistics and their cut-off scores: non-normed fit index (NNFI ≥ 0.9 for acceptable fit), comparative fit index (CFI ≥ 0.9 for acceptable fit), the root mean square error of approximation (RMSEA ≤ 0.1 for acceptable fit), and the standard root mean square residual (SRMR ≤ 0.1 for acceptable fit) (Hooper et al., 2008; Kline, 2015).

In the second step of the analyses, preliminary analyses were examined including descriptive statistics, analysis assumptions, and correlations for the variables of the present study. The assumption of normality was checked based on kurtosis and skewness values and their decision criteria, namely, skewness and kurtosis scores ≤ |1| (Tabachnick and Fidell, 2013; Kline, 2015). A Pearson correlation analysis was conducted next to investigate the association between suffering, life satisfaction, psychological adjustment, and self-transcendence. Afterward, we conducted a moderated mediation analysis to understand the protective role of self-transcendence on the mitigating effect of life satisfaction in the association between coronavirus-related suffering and psychological adjustment problems. The PROCESS macro version 3.5 (Hayes, 2018) was used to employ the moderated mediation model (see Figure 8) with the bootstrapping method with 10,000 resamples to estimate the 95% CIs (Preacher and Hayes, 2008; Hayes, 2018). The bootstrapping procedure is suggested to examine the significance of the indirect effect (Hayes, 2018). All analyses in the present study were conducted using SPSS version 25 (IBM, Armonk, NY) and LISREL version 8.8 (Scientific Software International Inc., Skokie, IL).

## Results

### Validity and Reliability Analyses of the STM

The results from the exploratory factor analysis, which was carried out using a principal component analysis with Promax (oblique) rotation, indicated that the measure yielded a two-factor solution with eigenvalues >1 that explained ~58% of the variance and was characterized by a lack of singularity (Bartlett's χ^2^ = 854.55, *df* = 45, *p* < 0.001) and an adequate sample size [Kaiser–Meyer–Olkin (KMO) = 0.88]. The factor loadings of the measure were adequate-to-strong, ranging between 0.4 and 0.93. Following that, the confirmatory factor analysis results affirmed the two-factor measurement model, providing adequate data-model fit statistics [χ^2^ = 90.89, *df* = 34, *p* < 0*.0*01, NNFI = 0.92, CFI = 0.93, RMSEA (95% CI) = 0.097 (0.073,0.12), SRMR= 0.062]. The STM also had adequate-to-strong factor loadings (λ range = 0.44-0.69), characterized by adequate latent construct (*H* = 0.75–0.77–0.86) and internal reliability estimates (α = 0.73–0.76–0.84), as seen in Figure 9. Additionally, the correlation results with the measures in this study provided further evidence for the concurrent validity of the measure. These results suggested that the STM is a reliable and valid measure for use in assessing the self-transcendence of people.

### Testing the Moderated Mediation Model

We first examined descriptive statistics for the variables of the study, as shown in Table 3. Descriptive statistics indicated that the kurtosis and skewness scores of the study variables ranged from −0.82 to 0.59, indicating that the measures of this study had relatively normal distribution (Tabachnick and Fidell, 2013; Kline, 2015). The correlation analysis results further revealed that coronavirus-related suffering was significantly and negatively associated with life satisfaction (*r* = −0.22, *p* < 0.001), while it positively correlated with psychological adjustment problems (*r* = 0.5, *p* < 0.001). However, the correlation of coronavirus-related suffering with self-transcendence was non-significant (*r* = −0.01, *p* = 0.907). Self-transcendence had a significant and positive correlation with life satisfaction (*r* = 0.33, *p* < 0.001) and a negative association with psychological adjustment problems (*r* = −0.2, *p* < 0.001). There was also a significant and negative correlation between life satisfaction and psychological adjustment problems (*r* = −0.47, *p* < 0.001), as seen in Table 3.

**Figure 10:**
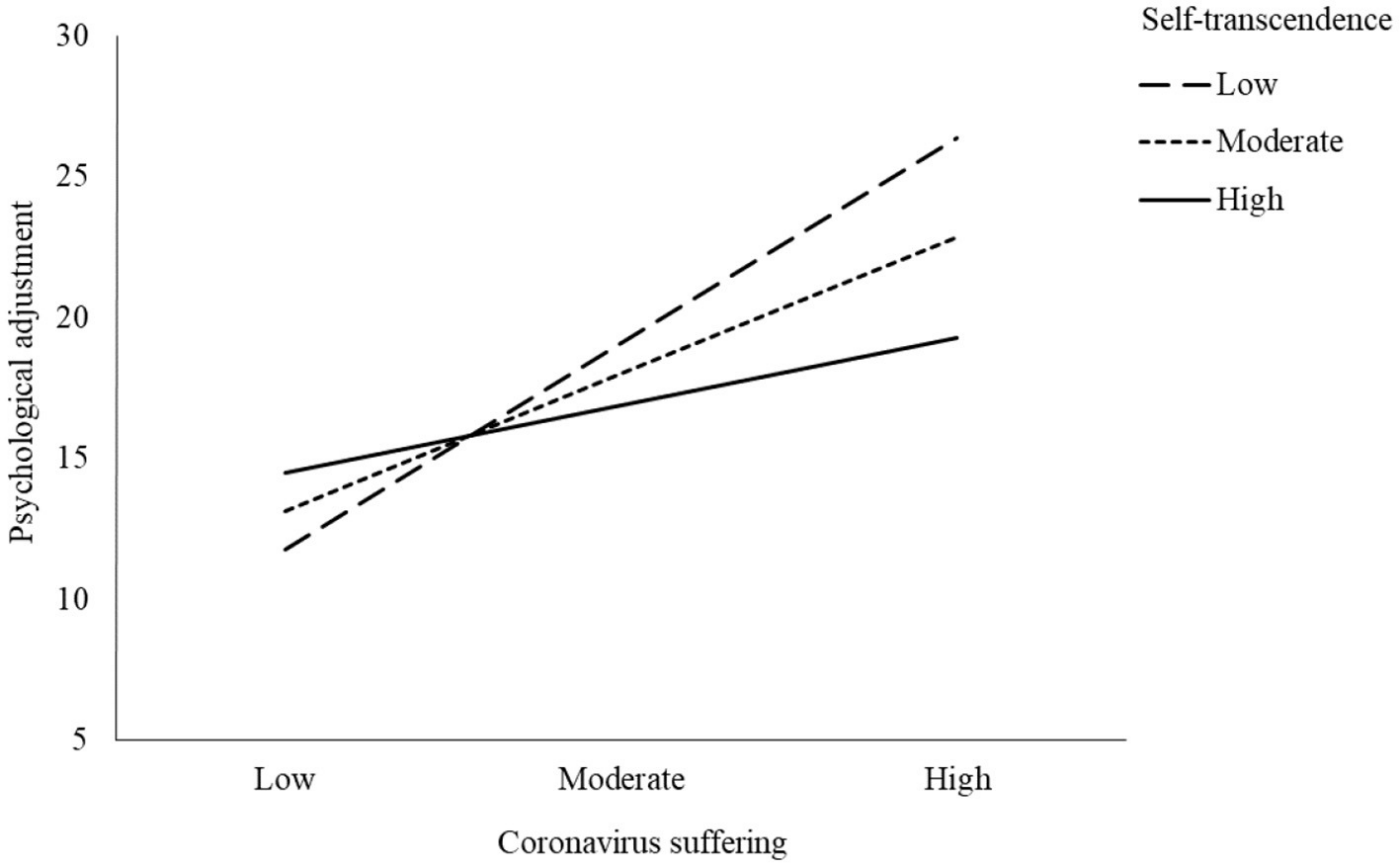
Moderating effect of self-transcendence on the link between coronavirus-related suffering and psychological adjustment problems.

Second, we conducted the moderated mediation model to test the protective role of self-transcendence on the mitigating effect of life satisfaction on the association between coronavirus-related suffering and psychological adjustment problems. The findings from the moderated mediation model showed that coronavirus-related suffering had a significant predictor effect on the life satisfaction of people (*b* = −0.2, *p* < 0.001) and accounted for 5% of the variance in this variable. Psychological adjustment problems were significantly predicted by coronavirus-related suffering (*b* = 0.66, *p* < 0.001) and life satisfaction (*b* = −0.45, *p* < 0.001). These results indicated that life satisfaction mitigated the adverse impacts of coronavirus-related suffering on the psychological adjustment of people. Further, the analyses demonstrated that the interaction between coronavirus-related suffering and self-transcendence on psychological adjustment problems was significant (*b* = −0.07; *F* = 16.71*, p* < 0.001), accounting for 5% of the additional variance in the model, as shown in Table 4. Overall, the model explained 46% of the variance in psychological adjustment problems. Moreover, the simple slope effect revealed that the indirect effect of coronavirus-related suffering on psychological adjustment was observed when self-transcendence was high (+1 *SD*), moderate, and low (−1 *SD*), as seen in Figure 10. These results indicate the protective effect of self-transcendence on the psychological adjustment of people during the coronavirus public health crisis.

**Table 4:** Unstandardized coefficients for the moderated mediation model.

**Consequent**								
		***M*** **(Life satisfaction)**				***Y*** **(Adjustment)**		
**Antecedent**		**Coeff**.	***SE***	***p***		**Coeff**.	***SE***	***p***
*X* (Coronavirus suffering)	*a*	−0.20	0.09	<0.001	*c*'	0.66	0.08	<0.001
*M* (Life satisfaction)		–	–	–	*b*_1_	−0.45	0.09	<0.001
*W* (Self-transcendence)		–	–	–	*b*_2_	−0.22	0.11	0.057
*X × W*		–	–	–	*b*_3_	−0.07	0.02	<0.001
Constant	*i*_M1_	24.26	0.45	<0.001	*i*_y_	28.94	2.36	<0.001
		*R*^2^ = 0.06				*R*^2^ = 0.46; *R*^2^ change = 0.05		
		*F* = 10.74; *p* < 0.001				*F* = 34.95; *p* < 0.001		
Indirect effects of coronavirus suffering on psychological adjustment								
	**Coeff**.		**BootSE**		**BootLLCI**		**BootULCI**	
Life satisfaction	0.09		0.04		0.03		0.17	

## Discussion

The purpose of this study was to test a moderated mediation model examining whether life satisfaction mediates the association between suffering during the pandemic and psychological adjustment problems and whether self-transcendence moderates this association. The results showed that coronavirus suffering was a significant predictor of life satisfaction and psychological adjustment problems, and life satisfaction mitigated the effect of suffering on psychological adjustment. Most importantly, self-transcendence is the mediating effect of life satisfaction on the association between suffering and adjustment. Self-transcendence served as a buffer against coronavirus suffering and promoted the adaptive psychological adjustment of people. Recently, the American Psychological Association (2020b) reported a nationwide mental health crisis in 2020 because of COVID-19. However, there is still a silver lining in the dark cloud ahead of us, that is, the prolonged lockdown and social isolation also provide a rare opportunity for self-reflection and self-transformation. More specifically, we suggest that learning to accept the current condition and re-orienting our own values and life goals toward something greater than ourselves can elevate our lives to a higher plane. The research of Emmons (1986, 2003) on Personal Goal Striving Theory can also account for the connection between self-transcendence and well-being because some of the worthy life goals, such as spirituality and generativity, are clear examples of self-transcendence. Meaning research has demonstrated that self-transcendence is a source of well-being as we reviewed in the introduction. Future research will clarify the importance of self-transcendence in all kinds of happiness, including an attunement with oneself, with others, and the world as a fundamental state of happiness and well-being (Haybron, 2013; Wong, 2014b).

We suggested that the STM represents an important instrument in advancing second-wave positive psychology (PP 2.0; Wong, 2011; Ivtzan et al., 2015) for several reasons. Firstly, it measures a sustainable well-being that is less dependent on positive emotions and positive circumstances. Secondly, it shifts the focus from behavior and cognition to the spiritual dimension that really separates human beings from other animals. Thirdly, it acknowledges that self-transcendence is the most promising path to moral virtue, eudaimonic happiness, and existential meaning. Frankl wanted to make sure that a good theory of self-transcendence needs to pass the Hitler test, that is, someone like Hitler can never lay claim to having lived a worthy self-transcendent life. This is why he was at pains to emphasize the conscience test, objective values, and ethical responsibility toward others. Similarly, Levinas (1972/2003) also emphasized that a fully functioning human will transcend self-interest in order to be ethically responsible for the Other.

To attain self-transcendence is our crowning humanistic and spiritual achievement, which enables us to move from instinctive selfishness to a state of selflessness and a higher level of consciousness. Self-transcendence motivates people to devote their time and energy to make positive contributions in society and appreciate little miracles in everyday life, thus resulting in greater well-being for themselves and others. Costin and Vignoles (2020) reported that a sense of mattering consistently emerged as a significant precursor of meaning in life. Mattering refers to the belief that the actions of an individual have made a difference in the world and that they have lived a significant life. We propose that such a belief is more based on living a life of self-transcendence, as measured by the STM-B.

It has taken more than 5 years, beginning with Wong (2016c), and involved many researchers from different countries to develop a reliable and valid measure of self-transcendence. Consistent with the literature (for examples, see Wong, 2014c; Yaden et al., 2017; Kaufman, 2020), our series of validation studies have demonstrated that the STM-B as a measure of or-orientation our focus and values away from egotism toward others and higher ideals s indeed significantly connected with meaning, benevolent values, virtues, eudaimonia, and mature happiness.

More importantly, we have demonstrated that the STM-B is a buffer against COVID-19 suffering. Thus, we propose that self-transcendence may be a unique antidote to adversity and suffering and a pathway toward personal growth and mature happiness. We hope that the STM-B can be used widely as an instrument to monitor global well-being in the era of the coronavirus pandemic. In sum, the concept of self-transcendence proposed by Frankl added both depth and breadth to meaning in life and may be an important breakthrough in mental health because it restores the soul or spiritual dimension as the healthy core in overcoming suffering and mental illness.

We realized the limitations of not relying more on prospective or longitudinal studies. Other recent research in the special issue of Frontiers on Self-Transcendence (Wong et al., 2021) has remedied this deficiency. On the positive side, it provided a useful instrument to study self-transcendence as a spiritual motivation or value.

Everyone is free to pursue what they believe will make them happy, but they are not free from the negative consequences of their choices. Often, the consequence could be the painful regret of hurting their loved ones or getting punished for their unethical means to achieve their egotistic ends.

This study showed that, by re-orienting the focus and values of an individual from their pre-occupation with happiness as their main life goal to selflessly striving toward a worthy life goal that benefits society, an individual is given a sense of satisfaction from the intrinsic value of pursuing meaning self-transcendence. Interestingly, some Japanese psychologists have developed a Fear of Happiness Scale and Fragility of Happiness Scale (Namatame et al., 2021). Such a fear is warranted in view of the avoidable sufferings from the blind pursuit of happiness. We hope that the STM-B will contribute to the global well-being through the less-traveled path of self-transcendence.

## Data Availability Statement

The raw data supporting the conclusions of this article will be made available by the authors, without undue reservation.

## Ethics Statement

The studies involving human participants were reviewed and approved by Trent University. The Ethics Committee waived the requirement of written informed consent for participation.

## Author's Note

PW was part of a working group of philosophers, theologians, and psychologists wrestling with the topic of virtue, happiness, and meaning. Given the absence of a general psychological theory and a valid measure of self-transcendence, the project by PW in that research working group was to fill this void. All of the presentations made by PW were first posted on a personal website. Only one presentation was published as a journal article; the rest of the materials were further developed and published for the first time.

## Author Contributions

PW and GA contributed to the design of the study. GA analyzed the data and wrote the method and results sections. PW wrote the introduction and discussion sections. All authors contributed to manuscript revisions, read, and approved the final version of the manuscript, agreed to be accountable for the content of the work, and approved the submitted version of the article.

## Funding

The study was partially supported by a 3-year Templeton Grant (https://www.templeton.org/grant/virtue-happiness-and-the-meaning-of-life-2).

## Conflict of Interest

The authors declare that the research was conducted in the absence of any commercial or financial relationships that could be construed as a potential conflict of interest.

## Publisher's Note

All claims expressed in this article are solely those of the authors and do not necessarily represent those of their affiliated organizations, or those of the publisher, the editors and the reviewers. Any product that may be evaluated in this article, or claim that may be made by its manufacturer, is not guaranteed or endorsed by the publisher.

## Appendix

### Self-Transcendence Measure-Brief (STM-B)

Please respond to the following statements by circling the most appropriate response to the scale, from 0 (not at all characteristic of me or my beliefs) to 4 (a great deal characteristic of me or my beliefs).

**Table:**

1.	My life is meaningful because I live for something greater than myself.
2.	My suffering is more bearable when I believe that it is for my family, friends, and/or for a higher purpose.
3.	I enjoy the process of striving toward excellence in what matters.
4.	At my funeral, I want to be remembered as a decent human being who cared about others.
5.	A worthy lifelong pursuit ought to have some intrinsic value—something that is good in its own right.
6.	What matters most to me in life is the contribution I make to society.
7.	I focus on discovering the potential meaning in every situation.
8.	I devote my life to pursuing the ideals of beauty, goodness, and truth.
9.	I develop my full potential in order to give my best to benefit society.
10.	I am more motivated by doing something meaningful than by the prospect of receiving external rewards.

For the original Self-Transcendence Measure (STM) (Wong et al., 2016), please see: Wong (2016c). For the Self-Transcendence Measure-Revised (STM-R) (Wong and Reilly, 2017), please see: Wong (2016d).
